# Long-term follow-up evaluation of readmissions after ventricular assist device implantation: trends and outcomes

**DOI:** 10.3389/fcvm.2026.1634771

**Published:** 2026-01-27

**Authors:** Mehmet Aksüt, Mustafa Mert Özgür, Hakan Hancer, Fatih Yigit, Ayhan Güneş, Kamile Topcu, Barış Gurel, Tanıl Özer, Özge Altaş, Sabit Sarıkaya, Kaan Kırali

**Affiliations:** Department of Cardiovascular Surgery, Kosuyolu High Specialization Research and Training Hospital, Istanbul, Türkiye

**Keywords:** HeartMate, LVAD, outcomes, readmission, VAD, ventricular access device

## Abstract

**Introduction:**

With the increasing utilization of left ventricular assist devices (LVADs) as a pivotal treatment option for end-stage heart failure, the rehospitalization of patients equipped with these devices has emerged as a significant issue impacting both quality of life and healthcare costs. This study evaluates readmission trends, predisposing factors, and their effects on survival over a long-term follow-up period for patients undergoing LVAD implantation.

**Methods:**

The study included 141 patients who underwent LVAD implantation between 2015 and 2023 and were followed for a minimum of 12 months. We analyzed the reasons for readmission, trends related to the devices, and overall outcomes.

**Results:**

The median duration of LVAD support was 49 months (IQR: 22–60). Overall, 102 patients (72.3%) experienced at least one readmission, with a median of two readmissions per patient (IQR: 0–3). Patients who were readmitted had a significantly higher body mass index (median 26.3 kg/m^2^ vs. 23.4 kg/m^2^, *p* = 0.003). In the multivariable Cox regression analysis, right ventricular dysfunction was the only factor independently associated with hospital readmission (HR = 1.769, 95% CI: 1.097–2.854, *p* = 0.019). Other variables—including body mass index, reoperative surgery, male gender, and tricuspid valve intervention—were not significantly associated with readmission. The most frequent causes of readmission were wound or driveline infections (33.7%), arrhythmias (16.9%), and right ventricular failure (11.8%). Long-term survival did not differ significantly between readmitted and non-readmitted patients (*p* = 0.335). Among device types, HeartMate III demonstrated the best survival outcomes [median 60 (40–60) months].

**Conclusion:**

Although LVAD implantation substantially improves survival in advanced heart failure, hospital readmissions remain common throughout long-term follow-up. Right ventricular dysfunction represents a key determinant of readmission risk, highlighting the importance of optimized perioperative management and vigilant monitoring for right-sided failure. Preventive strategies aimed at early detection of RV dysfunction and driveline complications may further reduce rehospitalizations and improve patient outcomes.

## Introduction

Heart failure remains one of the most significant health challenges today. Due to the shortage of heart transplant donors, coupled with the growing demand and advancements in medical technology, left ventricular assist devices (LVADs) have emerged as a crucial component in the treatment of end stage heart failure. The use of LVADs has been associated with a reduction in both mortality and morbidity in heart failure patients, while also improving their quality of life (1, 2).

With the growing use of left ventricular assist devices (LVADs) in recent years for both bridge-to-transplant (BTT) and destination therapy (DT) purposes globally, and the increased life expectancy following LVAD implantation, challenges related to LVAD follow-up have begun to surface. Complications such as infections, bleeding, and neurological issues have increasingly led to patient readmissions, raising concerns about the cost-effectiveness of this important therapeutic option for end-stage heart failure. Even though LVAD patients are followed closely and carefully by experienced clinics, these complications still remain the most important problem of the follow-up process.

In our study, we retrospectively examined a total of 141 patients with the most common reasons for readmission, emerging trends, and underlying causes in patients who were followed long-term after LVAD implantation.

## Methods

### Study design

In this comparative single-center study, 141 patients who underwent LVAD implantation and were followed for at least one year were retrospectively evaluated. We conducted a comprehensive analysis evaluating readmission trends, factors associated with readmissions, and clinical outcomes after LVAD implantation.

### Device-related analytical considerations

Although multiple LVAD types were included in the study, device type was not incorporated into the multivariable regression model. This decision was based on the unequal distribution of device groups, differences in implantation periods reflecting era-related effects, and the withdrawal of certain devices from the market during the study period. Including device type in the multivariable model under these conditions could have compromised model stability and interpretability. Therefore, device-specific outcomes and complication profiles were evaluated using descriptive and survival analyses rather than formal multivariable adjustment.

### Patients and inclusion criteria

Out of the 141 patients who underwent LVAD implantation 2015 and 2023, 141 individuals met the inclusion criteria for this study.

These criteria were as follows:

- Underwent LVAD implantation
- Followed for at least 12 months routinely in our clinic.

The exclusion criteria for this study were as follows:

- Patients who had heart transplantation within the first year after LVAD implantation
- Patients who are lost to follow-up or do not come for regular check-ups within the first year after LVAD.
- Patients who died in the perioperative period after LVAD other than right ventricular failure or a related cause

### Ethical considerations

Before initiating the study, ethical approval was obtained from the clinical research ethics committee of our hospital. We conscientiously adhered to the committee's directives and ethical benchmarks, primarily focusing on preserving patient privacy and confidentiality. The de-identification process meticulously eliminated any personally identifiable information from the dataset, ensuring absolute anonymity and enforcing rigorous data security protocols. These measures were instituted to provide the highest level of confidentiality for patient information, thereby facilitating a thorough and ethically sound investigation.

### Data collection

This study's data were meticulously gathered from various reliable sources. Extensive preoperative, postoperative and follow up information and was obtained by scrutinizing medical records from heart transplantation unit. Furthermore, these physical records were cross-checked with the national electronic health system to ensure precision.

### Preoperative evaluation

In our clinic, we follow the current guidelines and we assess patients with end-stage heart failure for suitability for LVAD implantation or heart transplantation through a multidisciplinary council. Prior to presenting cases to this council, we conduct a comprehensive evaluation that includes detailed echocardiographic reports, coronary angiography, right heart catheterization, cardiopulmonary exercise testing (if clinically appropriate), Electrocardiogram (ECG), chest x-ray, computed tomography (CT) of the lungs, brain, and abdomen, arterial and venous Doppler ultrasonography of the lower and upper extremities, carotid Doppler ultrasonography, abdominal ultrasonography, current laboratory tests, and preliminary psychological and neurological assessments.

### Operative approach

The implantation of LVADs was performed using either a standard median sternotomy or a minimally invasive approach, involving a left thoracotomy combined with a mini upper sternotomy through the third intercostal space. This approach was selected based on additional procedures performed on patients, the urgency of the procedure, patient preferences, or surgical planning. Initially, HeartMate II (HM2; Abbott, Chicago, IL, USA), HeartWare HVAD (HW; Medtronic, Minneapolis, MN, USA) and HeartAssist 5 devices were implanted; however, with the introduction of the HeartMate III (HM3; Abbott, Chicago, IL, USA) device to the market, the implantation of the HM3 device also commenced. Recently, with the withdrawal of the HW device from the market, the HM3 has become the sole device used in our clinic, as well as in many other clinics.

### Follow up and readmissions

In the postoperative follow-up, patients are routinely evaluated during outpatient clinic visits: one week after discharge, every two weeks for the first month, then at the third month, and subsequently at 3- to 6-month intervals depending on their condition and clinical course. During these visits, patients routinely undergo transthoracic echocardiography (TTE), ECG and laboratory tests including complete blood count (CBC), INR, lactate dehydrogenase (LDH), renal and liver function tests, brain natriuretic peptide (BNP) levels, and, if necessary, chest x-rays. Device parameters are checked and if necessary re-arranged according to clinical evaluation and results of the examinations. Patients are routinely asked whether they have experienced any emergencies or been hospitalized at another facility on these visits. Additionally, patients have 24/7 access to the LVAD coordinator via phone for urgent matters. When necessary, patient evaluations are also conducted through phone consultations. If hospitalization is required due to a medical issue, we prioritize and recommend admission to our own clinic; however, if the patient has been hospitalized elsewhere due to an emergency, we facilitate transfer to our clinic whenever possible. Readmission was defined as a hospital stay of at least one night.

Readmissions due to potential or actual heart transplants or false device alarms were excluded.

In the study, we categorized the reasons for readmission based on primary causes, which were classified as follows:

Driveline or wound site infections
Arrhythmias
Pulmonary complications (including causes such as pulmonary edema, pleural effusion, hematoma, etc.)
Gastrointestinal complications (e.g., melena, hematemesis, active rectal bleeding, as confirmed by the gastroenterology department)
Pump thrombosis (confirmed by CT angiography, elevated LDH levels, and pump parameters)
Right ventricular failure (confirmed by echocardiography and clinical evaluation)
Cerebrovascular infarction (confirmed by CT and neurological examination)
Hemorrhagic cerebrovascular event (confirmed by CT and neurological examination)
Anemia
Other causes (e.g., oral-nasal bleeding, petechial rash, etc.)

In the study, readmissions for potential or actual heart transplants, or false device alarms were excluded.

### Statistical analysis

All statistical analyses were performed using R Studio (v4.4.1, Boston, USA). The following R libraries were utilized: dplyr, ggplot2, survival, survminer, stats, MASS, car, rms, Hmisc, caret, pROC, nnet, and pscl.

Categorical variables were presented as frequencies and percentages, while continuous variables were expressed as mean ± standard deviation or median (interquartile range; 25th–75th percentiles), as appropriate.

Comparisons between two independent groups were performed using the Student's *t*-test for normally distributed data or the Mann–Whitney *U* test for non-normally distributed data.

Associations between categorical variables were assessed using the Chi-square test or Fisher's exact test, as appropriate.

Kaplan–Meier analysis was used to estimate survival and readmission-free survival probabilities, and survival curves were compared using the log-rank test. To evaluate factors independently associated with hospital readmission, a Cox proportional-hazards regression model was applied, incorporating variables with a univariate *p* value <0.20 into the multivariable analysis.

In addition, temporal trends in cumulative and annual readmission rates over the five-year follow-up were evaluated using the Cochrane–Armitage trend test. A *p*-value <0.05 was considered statistically significant for all analyses.

## Results

### Demographic characteristics

The study cohort consisted of 141 patients who underwent LVAD implantation, with a median age of 52 years (IQR: 42–57). Among them, 15 patients (10.6%) were female. Baseline clinical characteristics included hypertension in 43 patients (30.5%), diabetes mellitus in 52 patients (36.9%), and ischemic heart disease in 68 patients (48.2%). The median body surface area was 1.98 m^2^ (IQR: 1.82–2.16), and the median body mass index was 25.8 kg/m^2^ (IQR: 22.3–29). Additionally, 21 patients (15.3%) underwent reoperative surgical intervention (Table 1).

**Table 1:** Summary statistics of patient characteristics.

Variable	n (%)/Median (25th–75th percentile)
Age (years)	52 (42–57)
Gender (Female)	15 (10.6%)
Body Surface Area (m^2^)	1.98 (1.82–2.16)
Body Mass Index (kg/m^2^)	25.8 (22.3–29)
Hypertension	43 (30.5%)
Diabetes Mellitus	52 (36.9%)
Ischemic Heart Disease	68 (48.2%)
Re-op Surgery	21 (15.3%)
Readmission (Overall)	102 (72.3%)
Readmission Count	3 (2–4)

### Operative variables

**Figure 1:**
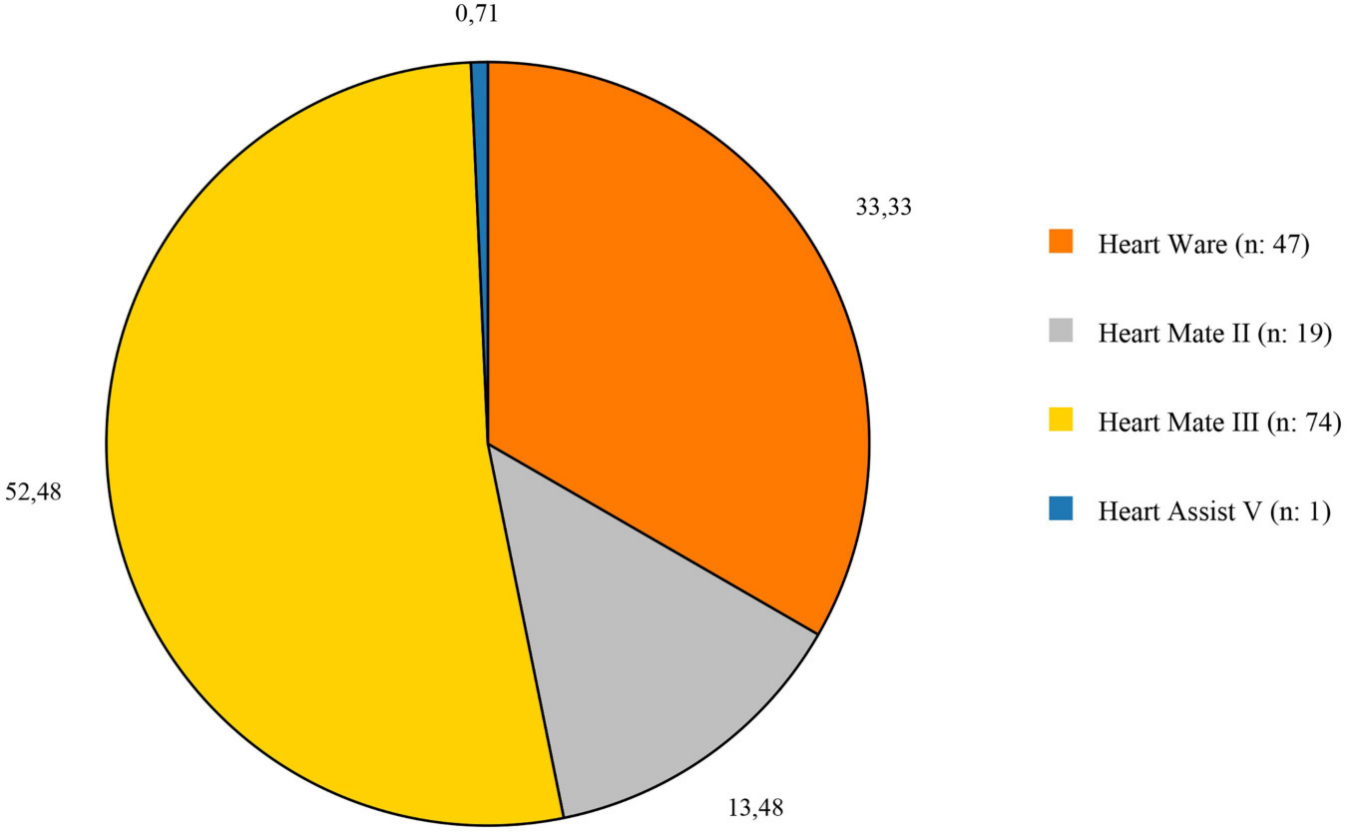
Distribution of the devices that used in the study.

HM3 was implanted in 74 patients (52.48%), making it the most frequently used device. HW, although it has now been withdrawn from the market, it was in active use at the time the study was conducted, implanted in 47 patients (33.33%) (Figure 1). In terms of surgical techniques, 56 patients (39.7%) underwent standard LVAD implantation with median sternotomy, while 37 patients (26.2%) received minimally invasive LVAD implantation. Concomitant procedures were performed in 28 patients (19.9%), and 13 patients (9.3%) had history of open heart surgery, with 6 of these also receiving concomitant procedures (Table 2).

**Table 2:** Operation types of patients.

Variable	n (%)
Standard LVAD	56 (39.7%)
Reoperative open heart surgery isolated LVAD	7 (5.0%)
LVAD + Concomitant Procedure	28 (19.9%)
Reop surgery- LVAD + Concomitant Procedure	6 (4.3%)
Minimally Invasive LVAD	37 (26.2%)
Minimally Invasive Redo LVAD	7 (5.0%)

LVAD, left ventricular assist device.

### Readmissions

During a median follow-up period of 49 months (IQR: 22–60), 102 patients (72.3%) experienced at least one readmission, with a median of 2 readmissions per patient (IQR: 0–3) and with a readmission rate of 1.68. The readmission rates among patients were as follows: 24.1% within the first 3 months, 22% between 3 and 6 months, 30.5% between 6 and 12 months, 50.4% within the first year, 33.3% between 1 and 2 years, 22.7% between 2 and 3 years, and 17.7% both between 3 and 4 years and between 4 and 5 years. There were a total of 237 readmissions after initial discharge in 102 patients. Comparative analysis between readmitted and non-readmitted patients revealed several significant differences. Patients who experienced readmission had a significantly higher body mass index (median 26.30 kg/m^2^ vs. 23.40 kg/m^2^, *p* = 0.003). Contrary to expectations, reoperative surgery was associated with a lower readmission rate (10.8% vs. 25.6%, *p* = 0.035). Other preoperative variables, including age, gender, hypertension, diabetes mellitus, and preoperative creatinine levels, did not show statistically significant differences between the readmitted and non-readmitted groups. The presence of concomitant procedures or the use of minimally invasive surgical techniques also did not significantly affect readmission rates (Table 3).

**Table 3:** Comparison of preoperative and operative variables based on readmission Status.

Variable	Readmission (−) (*n* = 39)	Readmission (+) (*n* = 102)	*p*-value
Gender (Male)	32 (82.1%)	94 (92.2%)	0.123*
Hypertension	11 (28.2%)	32 (31.4%)	0.715**
Diabetes Mellitus	13 (33.3%)	39 (38.2%)	0.589**
Ischemic Cardiomyopathy	18 (46.2%)	50 (49.0%)	0.761**
Reoperative Surgery	10 (25.6%)	11 (10.8%)	**0** **.** **035** **
Concomitant Procedure	10 (25.6%)	14 (13.7%)	0.115**
Minimally Invasive Operation	10 (25.6%)	34 (33.3%)	0.378**
Post LVAD RVD	1 (2.6%)	4 (3.9%)	1.000*
Age (years)	51 (41–54)	52 (43–58)	0.250
BSA (m^2^)	1.90 (1.79–2.00)	1.98 (1.83–2.13)	0.859
BMI (kg/m^2^)	23.4 (1.99–27.5)	26.3 (23.4–29.8)	**0** **.** **003**
Preoperative Creatinine (mg/dL)	1.04 (0.82–1.30)	1.01 (0.84–1.19)	0.544
Preoperative GFR (mL/min/1.73m^2^)	70.2 (47.3–100.9)	79.4 (64.7–95.9)	0.303

LVAD, left ventricular assist device; RVD, right ventricular dysfunction; BSA, body surface area; BMI, Body mass index; GFR, glomerular filtration rate.

Bold values refer statistically significant parameter.

* Fischer's exact test.

** Pearson chi-square.

### Factors associated with hospital readmission

In the Cox regression analysis evaluating factors associated with hospital readmission after LVAD implantation, right ventricular dysfunction was the only factor independently associated with readmission (HR = 1.769, 95% CI: 1.097–2.854, *p* = 0.019).

Other variables, including reoperative surgery (HR = 1.437, 95% CI: 0.742–2.782, *p* = 0.282), male gender (HR = 1.291, 95% CI: 0.620–2.690, *p* = 0.495), body mass index (HR = 0.976, 95% CI: 0.931–1.025, *p* = 0.335), and tricuspid valve intervention (HR = 1.604, 95% CI: 0.840–3.065, *p* = 0.153), were not significantly associated with hospital readmission (Table 4).

**Table 4:** Factors associated with hospital readmission after LVAD implantation (Cox regression analysis).

Variables	p	Odds ratio	95% confidence interval	
			Lower	Upper
Reoperative surgery	0.282	1.437	0.742	2.782
Gender(male)	0.495	1.291	0.620	2.690
Right Ventricle Disfunction	**0** **.** **019**	1.769	1.097	2.854
Body Mass Index (%)	0.335	0.976	0.93134	1.025
Tricuspit valve Intervention	0.153	1.604	0.840	3.065

Bold values refer statistically significant parameter.

### Causes of readmission

Analysis of readmission (Table 5) causes revealed that wound infections were the predominant reason for readmission (*n* = 80, 33.76%). Cardiac-related complications were also significant contributors, with arrhythmias being the second most common cause (*n* = 40, 16.88%), followed by right ventricular failure (*n* = 28, 11.81%). Pump thrombosis, a device-specific complication, represented (*n* = 27, 11.39%) of readmissions. Neurological complications, specifically cerebrovascular events, accounted for (*n* = 19, 8.02%) of cases. Gastrointestinal complications and other miscellaneous causes each constituted (*n* = 15, 6.33%) of readmissions. Less frequent causes included anemia (*n* = 9, 3.80%) and pulmonary complications (*n* = 4, 1.69%).

**Table 5:** Distribution of readmission causes.

Variable	n	%
Wound Infections	80	33.76%
Arrhythmias	40	16.88%
Pulmonary complications	4	1.69%
Gastrointestinal complications	15	6.33%
Pump thrombosis	27	11.39%
Right ventricular failure	28	11.81%
Cerebrovascular Event	19	8.02%
Anemia	9	3.80%
Other causes (e.g., oral-nasal bleeding, petechial rash, etc.)	15	6.33%
Total	237	100%

**Figure 2:**
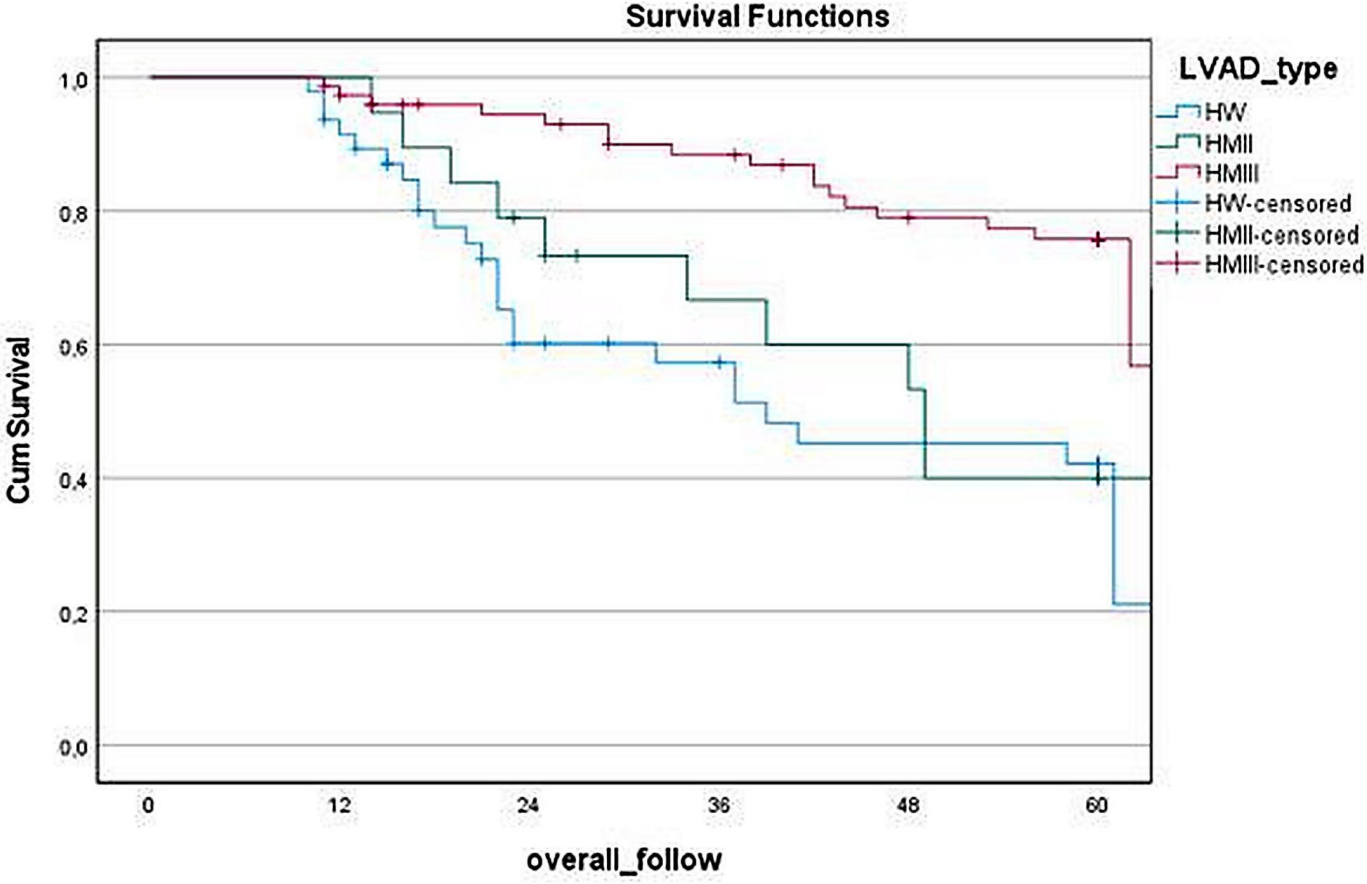
Kaplan–Meier survival curve based on LVAD types.

Figure 2 illustrates the survival analysis comparing different LVAD types, revealing significant differences in long-term outcomes (*p* < 0.05). HM3 demonstrated notably higher survival rates (75.7%) compare to both HW (42.2%) and HM2 (40.0%). The median survival duration was longest with HM3 60 (40–60) months, followed by HM2 39 (23–60) months and HW 23 (17–60) months.

**Figure 3:**
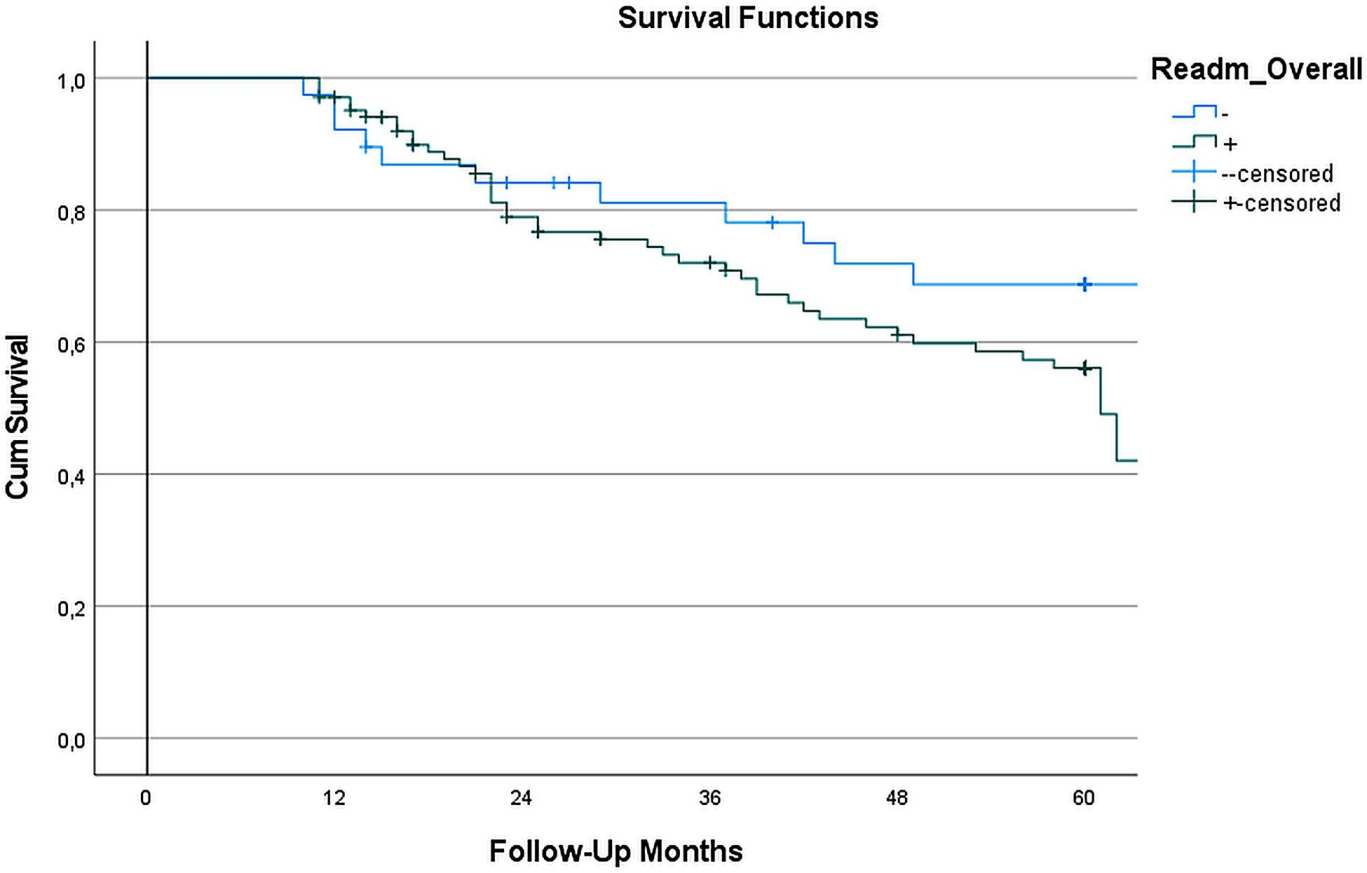
Survival analysis based on readmission Status: in the survival analysis of LVAD-implanted patients, kaplan–meier survival curves showed (Figure) no statistically significant difference between readmitted and non-readmitted groups during the 60-month follow-up. The log-rank test (Mantel-Cox) yielded a *χ*^2^ value of 0.928 (*p* = 0.335), indicating no significant difference in survival distributions between the two cohorts. The median survival time for the non-readmitted group was 55.1 months, while the readmitted group exhibited a median survival time of 56 months. These results suggest that readmission does not have a significant impact on long-term survival in LVAD patients.

Figure 3 presents the Kaplan–Meier survival analysis comparing patients with and without hospital readmissions after LVAD implantation. Over the 60-month follow-up period, we observed similar survival patterns between readmitted and non-readmitted patients (*p* = 0.335). The median survival times were comparable between groups, with non-readmitted patients surviving 55.1 (22–60) months and readmitted patients surviving 56 (26–60) months. These findings indicate that hospital readmission after LVAD implantation does not significantly impact long-term survival outcomes.

**Figure 4:**
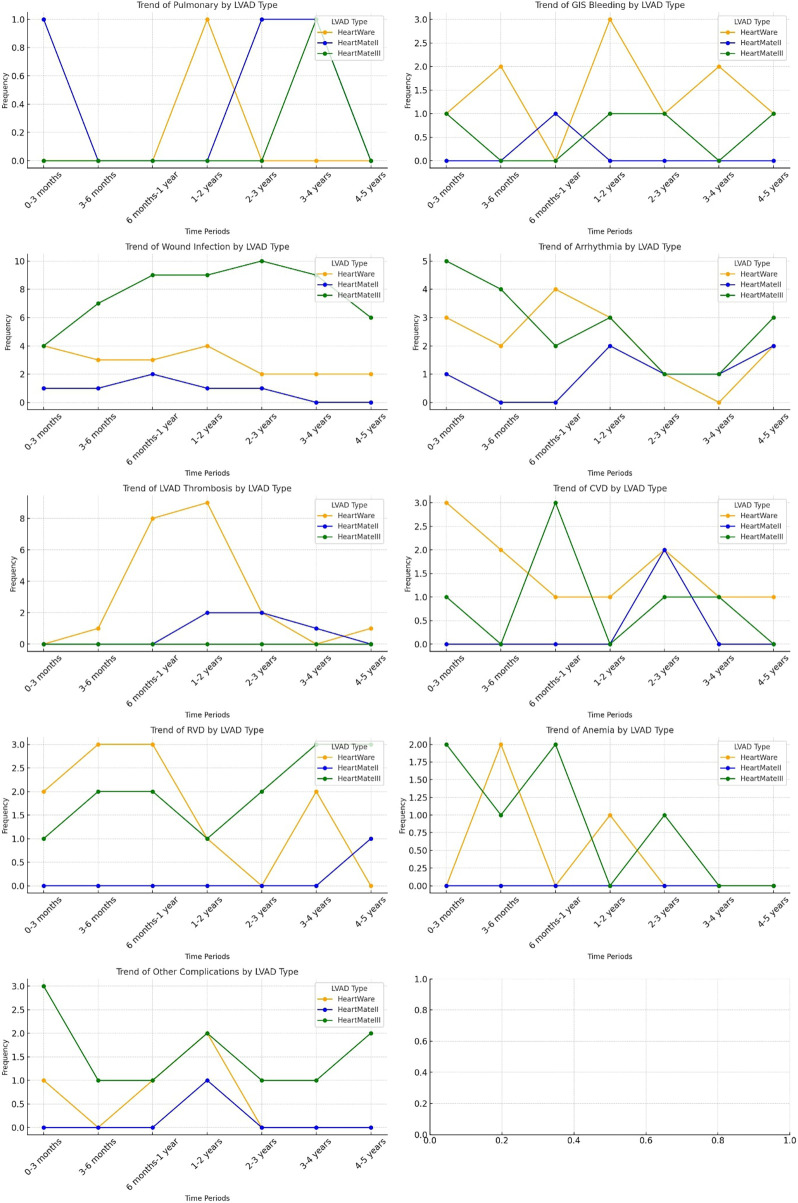
Distribution of readmission trends based on LVAD types.

Figure 4 illustrates the temporal distribution of readmission causes stratified by LVAD type throughout the follow-up period, providing a device-specific assessment of rehospitalization patterns. Distinct complication profiles were observed for each device. HeartMate III (HM3) recipients experienced a higher proportion of wound and driveline infection–related readmissions, particularly during the early postoperative phase, while arrhythmia-related readmissions markedly decreased after the first year of implantation. In contrast, HeartWare (HW) devices were predominantly associated with pump thrombosis–related readmissions, most notably between 6 months and 2 years following implantation. Cerebrovascular events and right ventricular dysfunction demonstrated moderate variability across device types, without a consistent temporal predominance. Pulmonary and gastrointestinal bleeding–related readmissions were relatively infrequent across all devices, with only minor variations observed among HM3 recipients. Anemia-related readmissions were more commonly observed in the HM3 group, particularly in the early postoperative period.

**Figure 5:**
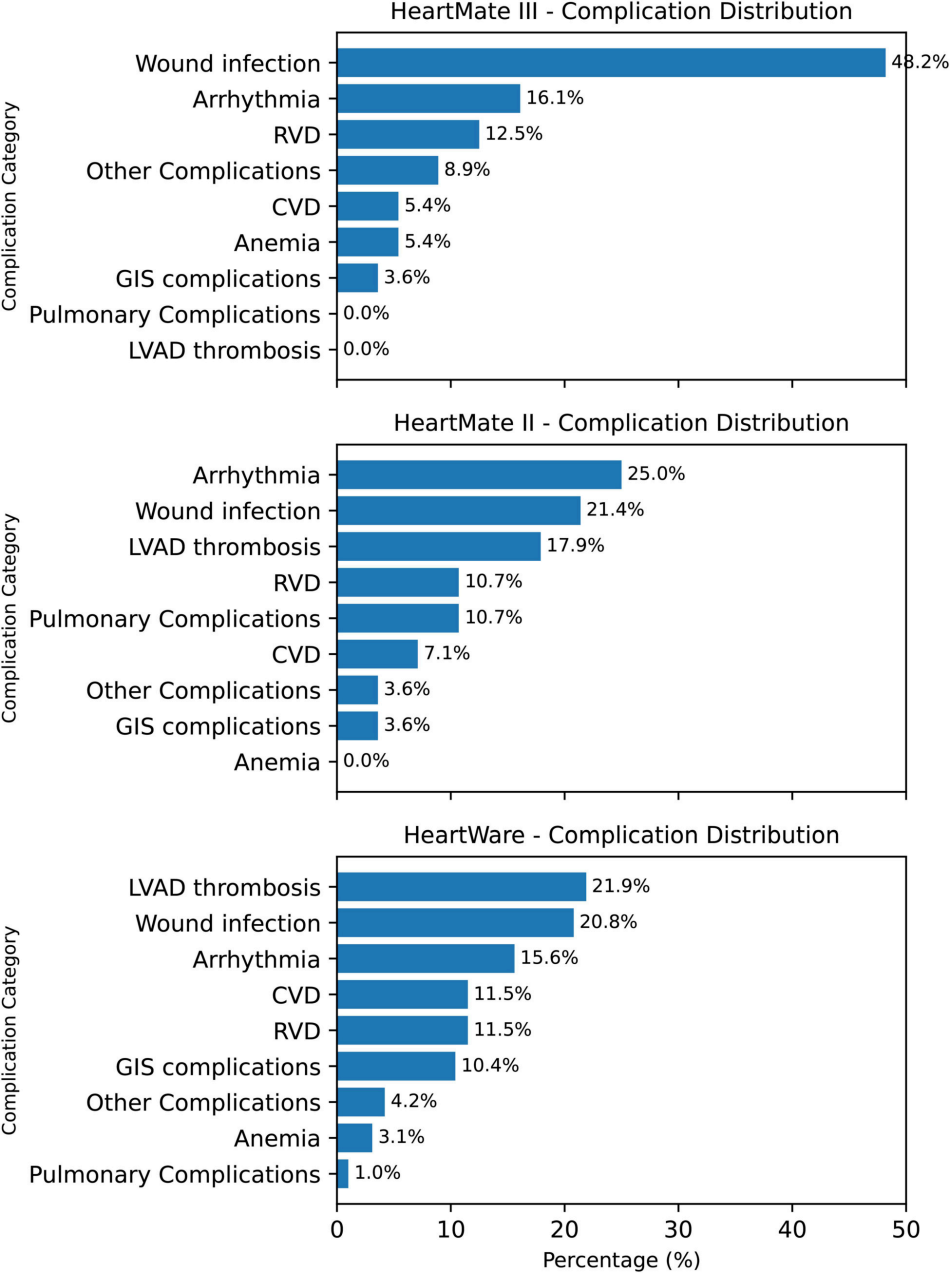
Device-specific complication profiles after LVAD implantation. Three horizontal bar panels (HM3, HM2, HW) display complication categories in descending order of frequency. HM3 is characterized by a high proportion of wound infection (48.2%); HM2 by arrhythmia (25.0%) with notable wound infection (21.4%) and pump thrombosis (17.9%); HW by pump thrombosis (21.9%) and wound infection (20.8%). Values indicate percent share within each device group.

Figure 5 further delineates device-specific complication profiles by presenting the relative distribution of major readmission causes within each LVAD subgroup. In the HeartMate III cohort, wound and driveline infections were the predominant cause of readmission (48.2%), followed by arrhythmias (16.1%) and right ventricular dysfunction (12.5%). Among HeartMate II recipients, arrhythmias constituted the most frequent cause of readmission (25.0%), followed by wound infections (21.4%) and pump thrombosis (17.9%). In the HeartWare subgroup, pump thrombosis (21.9%) and wound infections (20.8%) were the leading causes, with arrhythmias (15.6%) and cerebrovascular events/right ventricular dysfunction (each 11.5%) occurring less frequently. These findings highlight the heterogeneity of readmission etiologies across LVAD platforms and underscore that device-specific complication patterns were systematically evaluated and explicitly presented.

**Figure 6:**
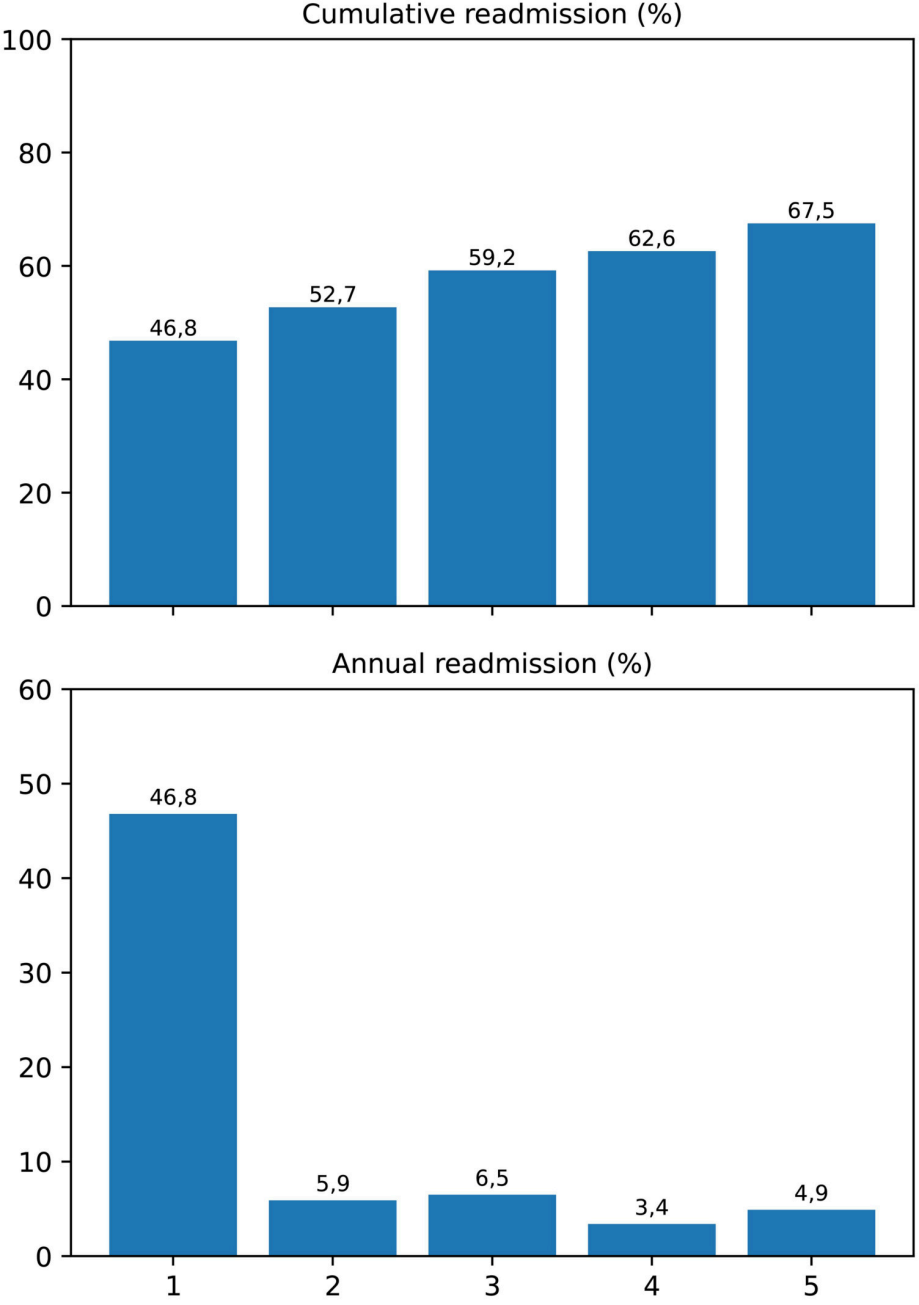
Cumulative and yearly incidence of hospital readmissions following LVAD implantation. The upper panel displays the cumulative percentage of patients readmitted within each postoperative year, while the lower panel illustrates the annual incidence rate. A significant decreasing linear trend was demonstrated by the Cochran–Armitage trend test (*p* = 0.002), indicating that most readmissions were concentrated within the first postoperative year.

Over the five-year follow-up, a marked concentration of readmission events was observed during the first postoperative year, followed by a gradual decline thereafter. The cumulative incidence of readmission increased from 46.8% at 1 year to 67.5% at 5 years, while the yearly incidence rate decreased from 46.8% to 4.9%. The Cochran–Armitage trend test confirmed a statistically significant decreasing linear trend in annual readmission rates (*p* = 0.002) (Figure 6).

## Discussion

In our study, 141 patients who underwent LVAD implantation and were followed for a median of 49 months (IQR: 22–60) were retrospectively examined.

One of the major treatment options for advanced heart failure, LVADs, are often associated with frequent readmissions during the post-implantation follow-up period, which in turn creates a significant financial burden (3, 4). Identifying the causes, temporal trends, and factors associated with readmissions during the follow-up period is crucial for improving patients' quality of life, reducing rehospitalizations, and mitigating the associated financial impact. For patients experiencing recurrent readmission episodes due to pre-implantation heart failure symptoms, which significantly impair their quality of life, it is an essential goal for heart failure clinicians to analyze, assess, and minimize these episodes following implantation, thereby also alleviating the burden on caregivers.

In our study, the median number of hospitalizations per patient, irrespective of device type, was 2, and our readmission rate was 1.68. These findings are consistent with those reported in the literature and indicate that, despite diligent efforts in LVAD patient management, a significant proportion—72.3%—of LVAD patients experience at least one readmission during the follow-up period for various reasons (5, 6). Additionally, as highlighted in the literature, readmission rates show a notably high incidence particularly within the first year following implantation, after which a gradual decline is observed (5). This trend may be attributed to both patient-related factors and factors such as patients lost to follow-up or those who have undergone heart transplantation. However, it can also be suggested that patient adherence to the device improves over a certain period.

When comparing patients with and without readmissions in terms of preoperative parameters, we observe that the BMI values are higher in the readmitted patient group. Similarly, in the study by Han et al., which investigated readmissions in patients undergoing LVAD implantation, increased BMI values were found to be significantly associated with higher rates of readmission (7). Moreover, studies by Mohamedali et al. and Brewer et al. also demonstrated that a BMI above 30 increases the frequency of readmissions (8, 9). Although patients with a high BMI appear to have a higher prevalence of comorbidities such as diabetes and ischemic heart disease, the study did not demonstrate a predisposing effect of these conditions on readmission.

When evaluating factors associated with hospital readmission, right ventricular dysfunction (RVD) emerged as the only variable independently associated with readmission in the multivariable Cox regression analysis (HR = 1.769, 95% CI: 1.097–2.854, *p* = 0.019). This finding underscores the importance of right-sided cardiac performance in maintaining post-LVAD clinical stability. RVD following implantation may contribute to recurrent hospitalizations through mechanisms such as venous congestion, suboptimal LVAD filling, and progressive end-organ dysfunction. Patients exhibiting early postoperative or borderline right ventricular function may therefore benefit from intensified hemodynamic monitoring and individualized optimization of preload and afterload conditions to prevent rehospitalization.

Other factors, such as reoperative surgery, male gender, body mass index, and tricuspid valve intervention, were not significantly associated with readmission risk. Although BMI was higher among readmitted patients in univariate analysis, this association did not persist after adjustment, suggesting that obesity may indicate general clinical complexity rather than act as an independent risk determinant. Our findings are consistent with previous reports showing that right ventricular dysfunction, rather than metabolic or surgical parameters, predominantly drives rehospitalization in LVAD recipients.

Meanwhile, driveline and wound infections remained the leading causes of readmission (33.7%), in line with prior studies emphasizing their recurrent nature and impact on long-term LVAD management (10, 11). Although not included as variables in the regression model to avoid conceptual overlap, these complications continue to represent a major clinical challenge. Strengthening infection-prevention protocols, improving patient education on driveline care, and early detection of local inflammatory changes remain key strategies for minimizing infection-related readmissions.

In our study, we found that infections, particularly driveline infections, are the leading causes of readmission among LVAD patients, aligning with findings by Vidula et al., who also identified these as the primary long-term readmission reasons in their work (5). Although some studies in the literature identify bleeding sharing the most common cause of readmission with infection, such issues are not frequently encountered in our clinic due to close monitoring of anticoagulation and antiplatelet therapy (12, 13). Of course, in cases where inevitable scenarios occur due to some pathologies such as angiodysplasia or acquired von Willebrand disease, readmission and further investigation may be necessary. However, minor bleeding events can be managed straightforwardly in an outpatient clinic, often with an interdisciplinary approach if needed without hospitalization.

In our study, while readmission in LVAD patients remains an unavoidably challenging issue, no significant difference in survival was observed between patients with and without readmission. Consistent with our findings, Han et al. and Akhter et al. reported no significant difference in survival in long-term follow-up between these two patient groups (7, 14). Conversely, Vidula et al. and Smedira et al. demonstrated significantly higher long-term mortality in patients who experienced readmission (5, 13). The underlying cause of these differing outcomes may be elucidated through further studies examining the rate of patients who underwent transplantation during the follow-up period, as well as the rate of patients lost to follow-up.

In device-based survival analysis, it is observed that HM3 demonstrates significantly better long-term survival rates (HM3 75.7%, HW 42.2%, and HM2 40.0%). The median survival duration was longest with HM3 [60 (40–60) months], followed by HM2 [39 (23–60) months] and HW [23 (17–60) months]. Many recent studies corroborate these findings, leading to the widespread adoption of HM3 as the most commonly used device (15, 16). However, it is important to note that there are still many patients with HW devices in various countries worldwide. To improve the treatment management of these monitored patients, uncover trends in readmission, and contribute to current knowledge, further research is needed.

In examining device-related readmission trends, we observe that infections account for over 20% of readmissions across all devices, with notably higher infection rates associated with HM3 devices. This trend, as previously discussed, is attributed to the significantly extended follow-up periods for HM3 patients, with infection-related hospitalizations being the primary cause of recurrent admissions. Additionally, while device thrombosis is observed as one of the most common causes of readmission in HW (21.9%) and HM2 (17.9%) devices, pump thrombosis is rarely seen in HM3 devices.

Although driveline and wound infections represented the most frequent causes of hospital readmission in our cohort, they were not independently associated with readmission in the multivariable analysis. This finding likely reflects the multifactorial and predominantly non-hemodynamic nature of infection-related hospitalizations, which are influenced by patient adherence, local wound care, environmental factors, and social determinants that are not fully captured by clinical regression models. In contrast, right ventricular dysfunction represents a systemic hemodynamic condition that may predispose patients to recurrent hospitalizations through venous congestion, impaired LVAD filling, end-organ dysfunction, and the need for repeated clinical optimization. Therefore, while infections account for the largest proportion of readmissions, right ventricular dysfunction appears to be more closely associated with the overall vulnerability to rehospitalization rather than with a single readmission etiology.

During the follow-up period, several measures were implemented in our clinic to reduce readmissions. In particular, the anticoagulation regimen was modified over time to minimize gastrointestinal and other bleeding events. Currently, we continue treatment with aspirin (ASA) 100 mg and warfarin, while maintaining INR levels between 1.7 and 2.2. Previously, a more aggressive anticoagulation strategy was adopted, targeting INR levels of approximately 2.5–3.0. In cases of recurrent or severe bleeding, after completion of the acute management period, some patients are maintained on warfarin monotherapy.

In addition, to minimize driveline infections, we have implemented more rigorous patient education protocols and revised our antibiotic therapy strategies. In particular, longer courses of antibiotic therapy are administered in resistant cases. We also place great emphasis on reducing traction at the driveline exit site and strongly advise patients to secure the driveline beyond the exit site and to remain vigilant regarding traction-related stress.

Although there are limited preventive measures available to completely avoid early or late right ventricular failure, patients are closely monitored with careful review of laboratory parameters and echocardiographic assessments, and device speed is optimized accordingly. Patients are also educated regarding appropriate fluid intake and urine output monitoring. When necessary, proactive measures are implemented.

### Limitations

Our study's single-center, retrospective design represents one of its primary limitations. Additionally, the study does not exclusively focus on patients undergoing pure destination therapy. Heart transplantation during follow-up due to donor availability at various time points also constitutes a limitation of the study. Conducting multi-center, prospective, randomized controlled trials with a broader population and long-term follow-up could contribute significantly to a better understanding of the reasons for readmission by examining all pathologies in greater detail.

A minimum follow-up duration of 12 months was applied as an inclusion criterion, which may be considered a limitation, as several LVAD-related complications can occur beyond the first postoperative year. However, it should be noted that the median duration of LVAD support in our cohort was 49 months (IQR: 22–60), indicating that the majority of patients were followed over a substantially longer period. This extended follow-up allowed for the assessment of both early and late readmission patterns and provides meaningful insight into long-term rehospitalization trends after LVAD implantation. Nevertheless, late-onset complications occurring beyond the observed follow-up window may not have been fully captured.

## Conclusion

In conclusion, our study indicates that the rates of readmission following LVAD implantation remain notably high throughout the follow-up period. However, it is observed that the trend of readmissions is particularly elevated in the first year and subsequently decreases in the following years. Additionally, it is evident that patients with infections and right ventricular failure serve as significant predisposing factors for recurrent readmissions. Although readmission and its associated factors negatively impact quality of life, no significant difference in long-term survival has been observed between patients who experience readmission and those who do not.

To reduce readmissions, significant progress can be achieved through the implementation of additional preventive measures against infections, close monitoring of anticoagulation and antiplatelet therapy to minimize bleeding, comprehensive and structured patient education, as well as future technological advancements.

## Data Availability

The raw data supporting the conclusions of this article will be made available by the authors, without undue reservation.
